# Cost-effectiveness of endovascular treatment for acute ischemic stroke in China: evidence from Shandong Peninsula

**DOI:** 10.1186/s13561-024-00513-7

**Published:** 2024-06-05

**Authors:** Lu Han, Kuixu Lan, Dejian Kou, Zehua Meng, Jin Feng, Elizabeth Maitland, Stephen Nicholas, Jian Wang

**Affiliations:** 1 Dong Fureng Institute of Economic and Social Development, Wuhan University, Wuhan, China; 2 Center for Health Economics and Management, School of Economics and Management, Wuhan University, Wuhan, China; 3 The Affiliated Hospital of Qingdao University, Qingdao, China; 4 School of Management, University of Liverpool, Liverpool, England, United Kingdom; 5 Health Services Research and Workforce Innovation Centre, Newcastle Business School, University of Newcastle, Newcastle, NSW Australia; 6 Australian National Institute of Management and Commerce, Australian Technology Park, Sydney, NSW Australia

**Keywords:** Cost-effectiveness, Endovascular treatment, Intravenous thrombolysis, Acute ischemic stroke, Direct treatment costs, Indirect costs

## Abstract

**Background:**

Recently, the endovascular treatment (EVT) of acute ischemic stroke has made significant progress in many aspects. Intravenous thrombolysis (IVT) is usually recommended before endovascular treatment in clinical practice, but the value of the practice is controversial. The latest meta-analysis evaluation was that the effect of EVT versus EVT plus IVT did not differ significantly. The cost-effectiveness analysis of EVT plus IVT needs further analysis. This study assesses the health benefits and economic impact of EVT plus IVT in Shandong Peninsula of China.

**Method:**

We followed a cross-section design using the Chinese-Shandong Peninsula public hospital database between 2013 and 2023. The real-world costs and health outcomes were collected through the Hospital Information System (HIS) and published references. We calculated incremental cost-effectiveness ratios (ICERs) from the perspective of Chinese healthcare using the complex decision model to compare the costs and effectiveness between EVT versus EVT + IVT. One-way and Monte Carlo probabilistic sensitivity analyses were performed to assess the robustness of the economic evaluation model.

**Results:**

EVT alone had a lower cost compared with EVT + IVT whether short-term or long-term. Until 99% dead of AIS patients, the ICER per additional QALY was RMB696399.30 over the willingness-to-pay (WTP) threshold of 3× gross domestic product (GDP) per capita in Shandong. The probabilistic sensitivity analysis of 3 months, 1 year and long-term horizons had a 97.90%, 97.43% and 96.89% probability of cost-effective treatment under the WTP threshold (1×GDP). The results of the one-way sensitivity analysis showed that direct treatment costs for EVT alone and EVT + IVT were all sensitive to ICER.

**Conclusions:**

EVT alone was more cost-effective treatment compared to EVT + IVT in the Northeast Coastal Area of China. The data of this study could be used as a reference in China, and the use of the evaluation in other regions should be carefully considered.

**Supplementary Information:**

The online version contains supplementary material available at 10.1186/s13561-024-00513-7.

## Background

Stroke is the second leading cause of death worldwide, the third leading cause of combined disability death and the number one cause of disability-adjusted life years (DALYs) in China [1]. As the most common type of cerebrovascular disease in China. ischemic stroke (IS) accounted for 83% of the hospitalized patients with cerebrovascular diseases in 2019, with an annual recurrence rate ranging between 9.6 and 17.7% [2–4]. The per capita hospitalization medical expenses for IS in China have shown an increasing trend since 2010, with the per capita hospitalization expenses for IS rising from RMB9824 in 2020 to RMB10740 in 2021, a year-on-year increase of 9.32% [5, 6].

With a therapeutic time window of 8 h for acute ischemic stroke (AIS) treatment, endovascular thrombolysis (EVT) and intravenous thrombolysis (IVT) treatments are regularly used clinically to remove the thrombus [7]. Recent Chinese clinical evidence suggests that EVT is more effective than intravenous thrombolysis (IVT) with less complications and lower mortality [8]. However, we should aware that EVT may cause ischemia - reperfusion (I/R) damage while restoring blood flow to the ischemic organ, with the risk of prolonging the prognosis of AIS. Appropriate treatment techniques are key for AIS patients to benefit fully from endovascular therapy, attenuate catastrophic health expenditures and economize on healthcare costs.

Diaz et al. evaluated mechanical thrombectomy (MT) as one way EVT can lead to better health outcomes per AIS patient and achieve cost savings from first pass effect (FPE), or complete/near revascularisation of the large-vessel occlusion [9]. Clinical evidence from China associates achieving FPE with good outcomes after MT surgery [10, 11]. Aronsson et al. showed adding thrombectomy with stent retrievers improved quality-adjusted life-years (QALY) and delivered cost savings of approximately US$221 per patient [12]. Recent studies of carotid endarterectomy (CEA) after EVT showed high long-term outcomes compared with IVT alone and standard care (SC) [13–15] and the proven long-term benefits of EVT always offset the high costs on surgery. In developing countries like China, the economic factor is one of the important concerns that patients and their families consider when choosing a treatment plan for chronic diseases [16, 17]. Therefore, it is important to evaluate the cost-effectiveness of EVT alone versus EVT + IVT for AIS patients over their lifetime in China.

## Method

### Model structure

**Fig. 1:**
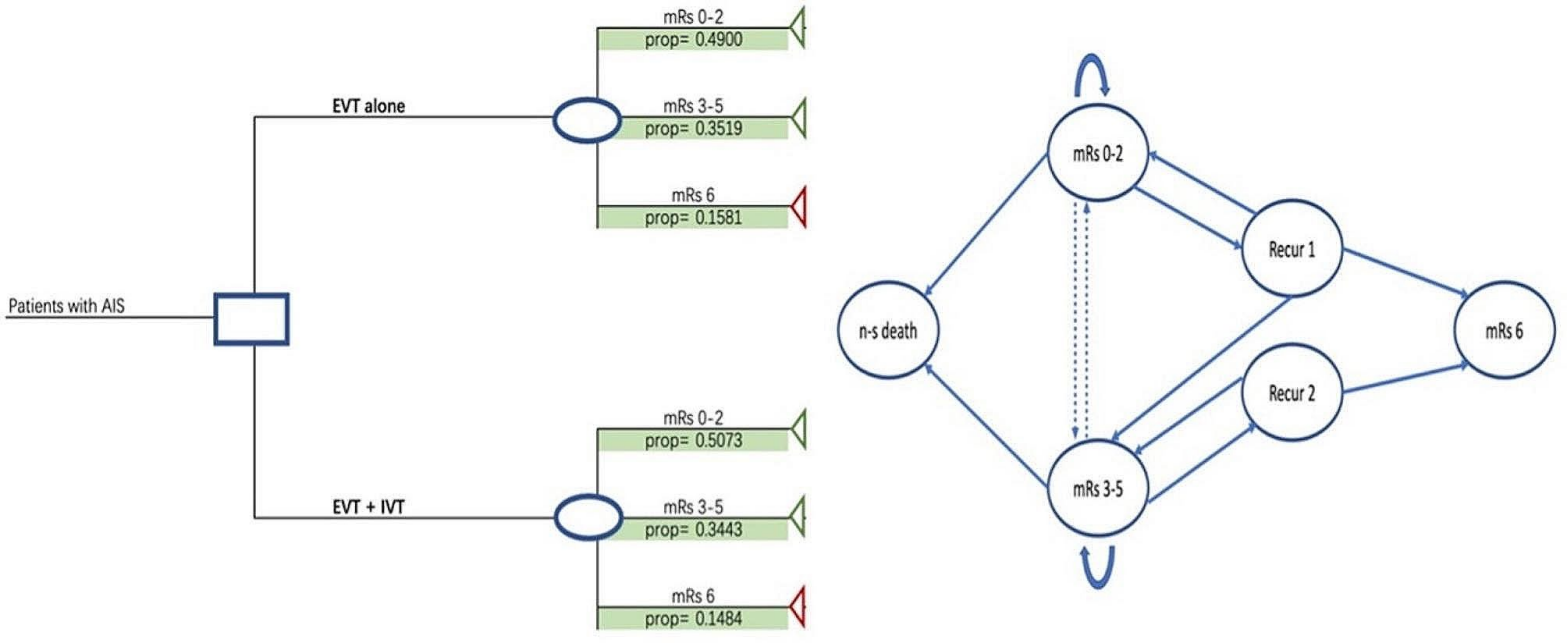
Decision model for EVT + IVT versus EVT alone Note: AIS, acute ischaemic stroke; mRS, modified Rankin Score

Figure 1 depicted the health economic decision model for AIS patients, comprising EVT alone versus EVT + IVT for their life horizon. In the first three months of their treatment, a decision tree model was designed and grouped by EVT alone versus EVT + IVT. For each treatment, we used the Chinese Modified Ranking scale (mRS) to define three states including good outcome (mRS 0–2); poor outcome (mRS 3–5); or death (mRS 6). We considered 90 days as the initial cycle, and then all survivors entered the Markov model until 99% of AIS patients died, using every three months as a cycle [18, 19]. AIS patients’ median age of EVT alone was 71 years (IQR 62–78) and EVT + IVT was 70 (IQR 62–78) [19]. The decision tree model plus the Markov state-transition model was conducted in Excel 2013 [20].

### Model input parameters and data

Based on a EVT-IVT meta-analysis of six randomised trials [19], we selected the proportions of patients in different mRS states at the end of 3 months and the recurrent rate at the baseline as shown in Table 1. We assumed the Markov model with the recurrent patients between mRS 0–2 and mRS 3–5 in the first year and the recurrent treatment pattern was the same as the first stroke treatment [21, 22]. After the first year, AIS patients could remain in their current state (mRS0-2 or mRS3-5), transfer to the recurrent state, or die due to the non-stroke causes every three months. The dependent patients remained in their health state or death and the probabilities of each health state in independent patients were the same as for the first occurrence [21].

**Table 1:** Model inputs in base case

Health outcome	EVT alone	EVT + IVT	Distribution	Parameters
Proportions of patients in different mRS states at the end of 3 months according the meta analysis (%)				
mRS 0–2	49.00 [19]	50.73 [19]	Dirichlet	0–1
mRS 3–5	35.19 [19]	34.43 [19]	Dirichlet	0–1
mRS 6	15.81 [19]	14.84 [19]	Dirichlet	0–1
Probabilities(%)				
Recurrent rate	9.60 [30]	9.60 [30]	Beta	/
mRS 0–2 to mRS 0–2 in first year	95.50 [21]	95.50 [21]	Dirichlet	0–1
mRS 0–2 to mRS 3–5 in first year	2.40 [21]	2.40 [21]	Dirichlet	0–1
mRS 3–5 to mRS 0–2 in first year	2.90 [21]	2.90 [21]	Dirichlet	0–1
mRS 3–5 to mRS 3–5 in first year	91.90 [21]	91.90 [21]	Dirichlet	0–1
Costs(RMB)				
Direct treatment costs	71329.86	108463.60	Gamma	EVT alone: 69890.76-72768.96 EVT + IVT: 86765.90-130161.30
Operative treatment	6543.83	10041.25		
Operative materials	43751.21	52879.26		
Medicine	6984.55	13285.07		
Nursing	844.83	3035.45		
Annual post-hospitalization costs				
mRS 0–2	7385.00 [23]	7385.00 [23]	Gamma	7157–7619
mRS 3–5	11350.00 [23]	11350.00 [23]	Gamma	10,730–11,996
Indirect costs	57595.37	57595.37	/	
DALYs	7.95 [22]	7.95 [22]	/	
Utility				
mRS 0–2	0.76 [25]	0.76 [25]	Beta	0.69–0.82
mRS 3–5	0.21 [25]	0.21 [25]	Beta	0.17–0.26
mRS 6	0.00 [25]	0.00 [25]	Beta	/
Recurrent stroke	0.20 [25]	0.20 [25]	Beta	0.16–0.26
Discount Rate(%)				
Annual post-hospitalization costs	3.00	3.00	/	
Indirect costs	3.00	3.00	/	
Outcome	3.00	3.00	/	

Note: RMB, Chinese yuan renminbi; mRS, modified Rankin Score; EVT, endovascular treatment; IVT, intravenous thrombolysis

The costs were calculated from the branches of Shandong Peninsula Top Three Public Hospital affiliated with Qingdao University. We designed a cross-sectional survey of ischemic stroke patients from January 2013 to September 2023 and calculated the mean and 95% confidence interval (CI) of direct treatment costs. First, a total of 42,554 patients with disease code I63 and its sub-classification were extracted from the 10-year hospital official database. Then a total of 3,449 patients with EVT (00.6 and its sub-classification, as well as 39.74) were extracted according to the primary surgical code, 30 patients with EVT + IVT whose operation code was I99.1 were selected according to the second operation code until the sixth operation code. The real-world treatment costs provided detailed data, including medicine costs, operative costs, operative materials costs, nursing costs and various associated costs. Indirect costs of combining the human capital approach with disability-adjusted life years (DALYs) were calculated in the base-case year. Since the HIS system could not monitor post-hospital costs associated with the diseases, we extracted the annual post-hospitalisation costs from the Chinese cost-effectiveness of mechanical thrombectomy study [23]. All the costs discounted to the 2023 RMB level at a discount rate of 3% per year [24].

We calculated Quality-adjusted life-years (QALYs) to get health outcomes by multiplying longevity with utility scores derived from the literature on the Chinese stroke population [25]. The utility score was developed using the European Quality of Life Scale-5 dimension (EQ-5D), and the Chinese preference weights as shown in Table 1. According to the Chinese pharmacoeconomic Evaluation guidelines [24], we considered the same discount rate of health outcomes over time as the cost.

### Statistical analysis

The incremental cost-effectiveness ratio (ICER) was calculated by dividing the difference in costs by the difference in QALYs between the two treatments. Since the willingness to pay (WTP) threshold has not yet been defined in China, we adopted the WHO recommendation, which was consistent with the Chinese pharmacoeconomic evaluation guidelines with 1–3 times gross domestic product (GDP) per capita as the threshold [24, 26]. When ICER was less than three times GDP, the strategy was defined as cost-effective. We use 2023 as the base-case year and the WTP threshold corresponds to RMB90620 /QALY to RMB271860 /QALY for Shandong Province [27].

### Sensitivity analysis

We conducted one-way sensitivity analysis and Monte Carlo probabilistic sensitivity analysis to verify the internal validity of the decision model [28]. Under the one-way sensitivity analysis, the robustness of the model was evaluated by changing the range of one parameter (± 20%), while keeping the other parameters fixed. A probabilistic sensitivity analysis was used to verify the random uncertainty of parameter changes simultaneously in the model. The probabilistic sensitivity analysis by using Monte Carlo simulation in Excel 2013 and set at 10,000 iterations.

Following the gamma distribution, the cost data were non-negative and right-skewed [29]. The utilities and recurrent rate as a continuous probability within the interval (0,1) followed the beta distribution. When mutually incompatible events occurred and the sum of probabilities was a constant 1, a Dirichlet distribution was usually assumed. Hence, the proportion of patients with different mRS states and the transferred probability followed the Dirichlet distribution [28]. The two different sensitivity analysis results were represented by the scatter plot and the tornado plot.

## Results

### The cost-effective analysis

We analyzed the ICER for the EVT versus EVT + IVT in the base-case, 1-year time horizon and long-term until 99% dead. We found that EVT alone was more cost-effective compared to EVT + IVT both in the short and long term. In the first year, EVT had 0.74 QALYs at a cost of RMB 82358.75 and EVT + IVT had 0.76 QALYs at RMB119568.88. In this case, the ICER for both treatments was RMB 2004182.77 /QALYs, which meant EVT + IVT was not cost-effective.

Until 99% dead, EVT gained 2.82 QALYs at a cost of RMB271740.90 compared to EVT + IVT, which earned 2.95 QALYs at a cost of RMB359160.29. In this case, the ICER for per additional QALY was RM696399.30 (> RMB 271,860 /QALYs). Table 2 shows the cost-effectiveness results for different time horizons. Compared to EVT + IVT, EVT had a lower cost-effectiveness ratio (RMB 96234.35 /QALYs).

### Sensitivity analysis

**Fig. 2:**
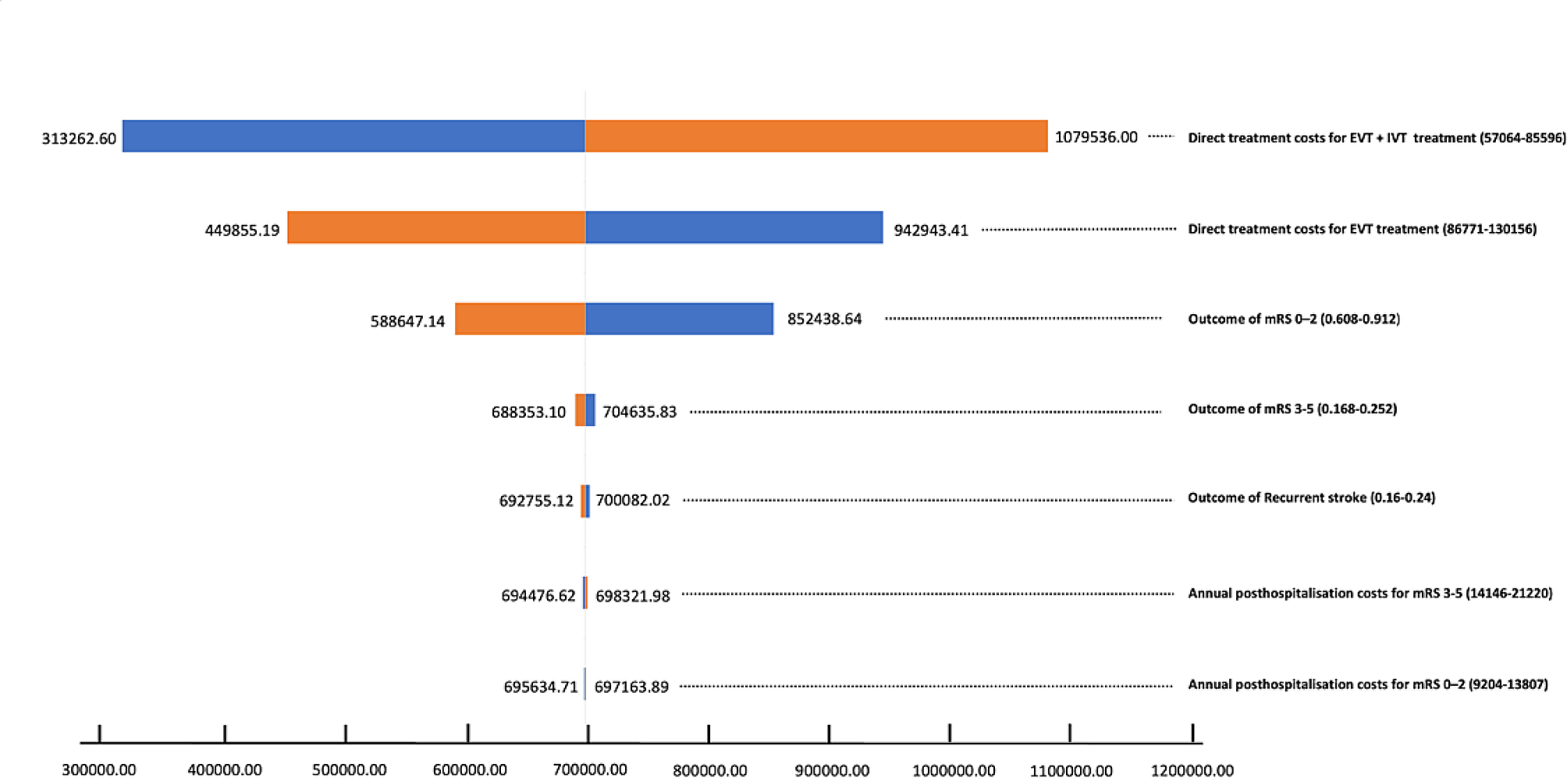
Tornado diagram of one-way sensitivity analyses. ICER, incremental cost-effectiveness ratio

Figure 2 presented the results of one-way sensitivity analysis using the tornado diagram. ICER was sensitive to both EVT and EVT + IVT direct treatment costs. When the direct treatment cost of EVT + IVT was reduced by 20%, the ICER (RMB313262.60/QALYs) was closer to the WTP (RMB90620 /QALYs-RMB 271,860 /QALYs), but that did not mean EVT + IVT was cost-effective. EVT was always more cost-effective than EVT + IVT, regardless of how other parameters varied within the range, which could also illustrate the robustness of the results.

**Fig. 3:**
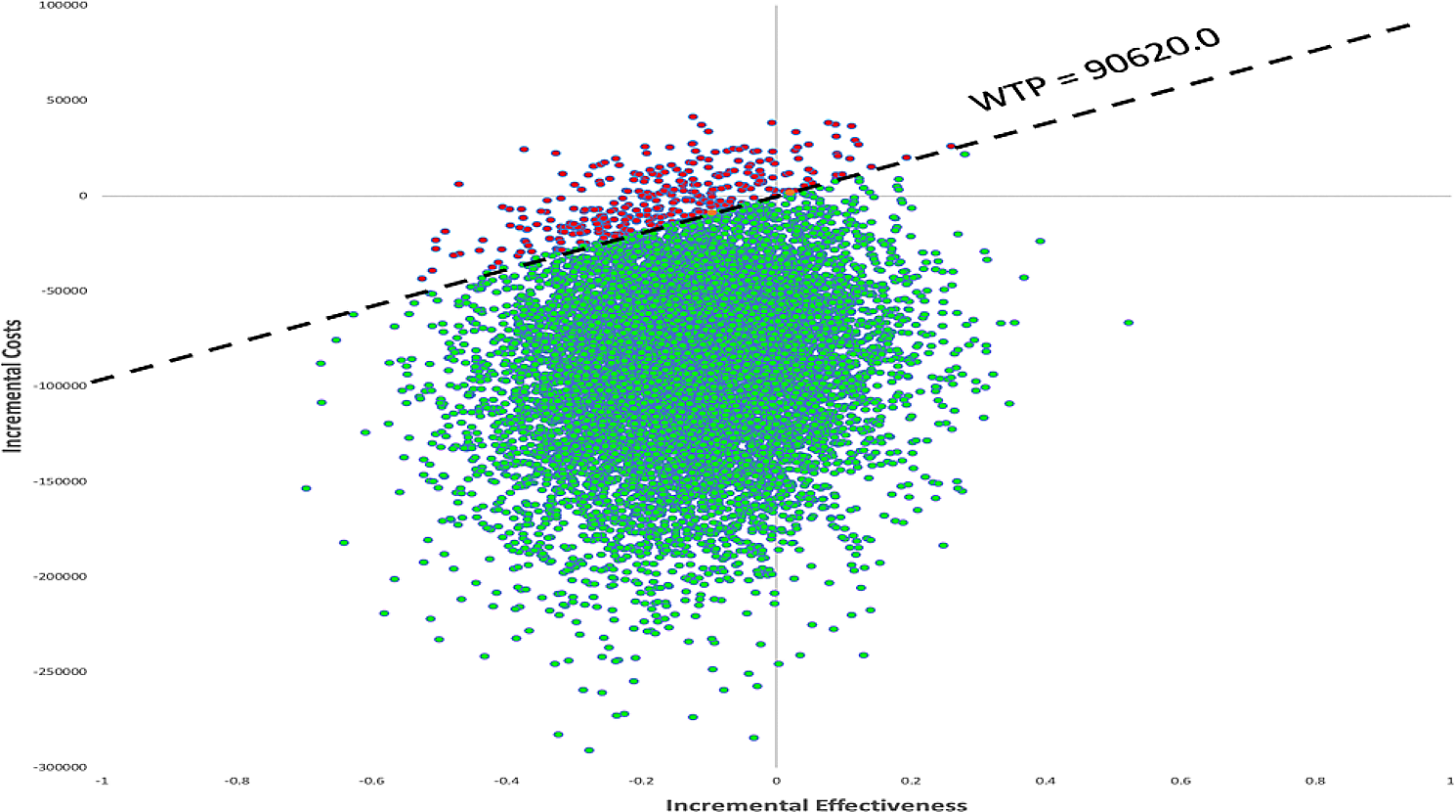
Scatter plot according to probabilistic sensitivity analysis until 99% of AIS death

**Fig. 4:**
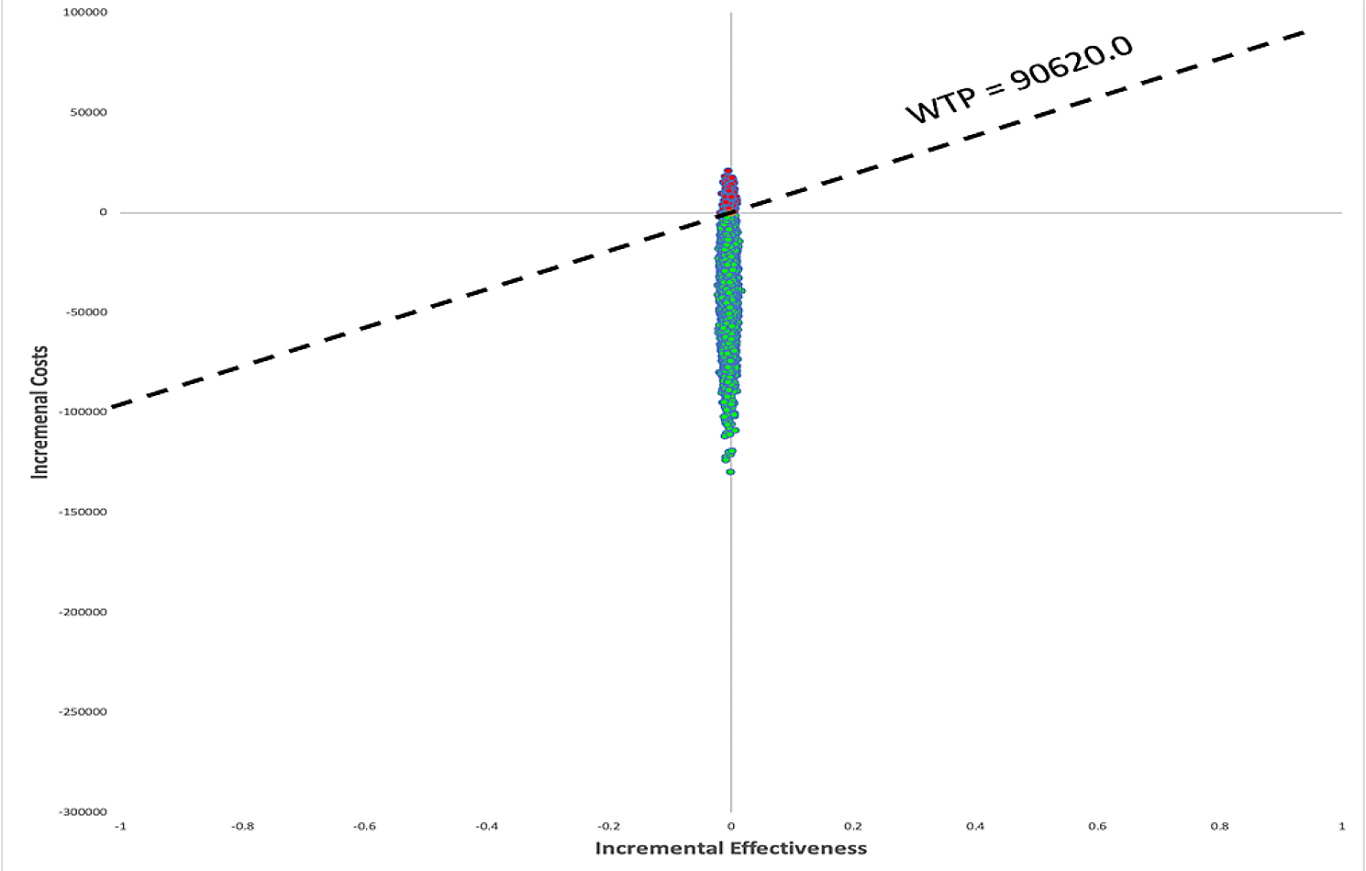
Scatter plot according to probabilistic sensitivity analysis for 3-month

**Fig. 5:**
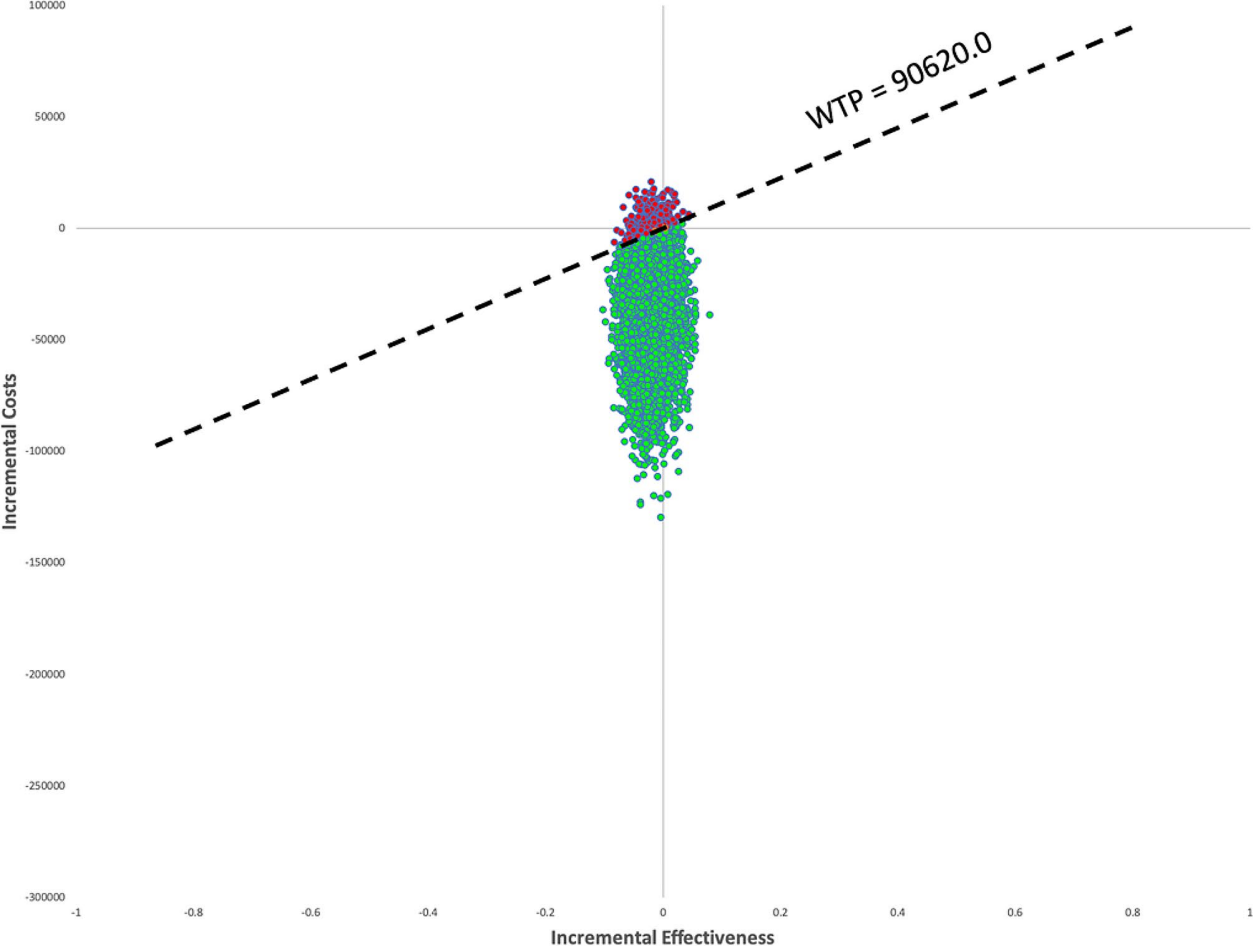
Scatter plot according to probabilistic sensitivity analysis for the first year

Figure 3 indicated the results of the probabilistic sensitivity analysis for cycling 10,000 times with parameters of the model inputs shown in Table 1 under the 99% of the AIS patient dead scenario. Compared with EVT + IVT, EVT alone was cost-effective in 96.89% of cases with the RMB 90,620 /QALYs threshold. From Figs. 4 and 5, we also found that EVT alone was a cost-effective treatment in 97.90% (3 months) and 97.43% (1 year) of cases under the threshold.

**Table 2:** The results of cost-effectiveness

Time horizon	Strategy	Cost	QALYs	Cost- effectiveness ratio	ICER
3 months	EVT alone	71329.86	0.15	461844.71	9288851.75
	EVT + IVT	108463.60	0.16	684558.18	
1 year	EVT alone	82358.75	0.74	111565.82	2004182.77
	EVT + IVT	119568.88	0.76	157998.14	
The life horizon	EVT alone	271740.90	2.82	96234.35	696399.30
	EVT + IVT	359160.29	2.95	121779.32	

Note: ICER, incremental cost-effectiveness ratio; QALYs, quality-adjusted life-years

## Discussion

We assessed the real cost-effectiveness of EVT alone and EVT + ITV for AIS and measured ICERs for life expectancy in Shandong Province, China, in 2023. Regardless of time horizons, EVT alone was a more cost-effective treatment compared with EVT + IVT for AIS. The direct treatment costs were derived from the HIS, while the indirect costs and utility values not in the HIS were derived from the published literature. ICER was more sensitive to the direct treatment costs when using EVT alone and EVT + IVT. Especially in low- and middle-income countries, the high cost of treatment has always been one of the most important reasons affecting stroke patients’ medical behavior [31–33].

Our study complemented work on AIS related topics in other countries. Mechanical thrombectomy (MT) after IVT was cost-effective in Argentina, reducing future disability and comorbidities over time [34]. Other studies in high-income countries also showed that MT was one way EVTs were a cost-saving treatment and improved QALY for AIS patients in the long term. For example, MT was performed at lower costs in a lifetime horizon versus thrombolysis in Chile, and with higher benefits (2.63 incremental QALYs) [35]. Related studies were also consistent in adding stent retriever thrombectomy, globally (0.73 incremental QALYs in France) [36]. American research showed adding thrombectomy with stent retrievers to guideline-based care (including IVT) resulted in a gain of 0.99 QALYs with a cost savings of approximately $221 per patient [12]. These previous studies had shown the benefits of mechanical thrombectomy.

In China, Han compared the mechanical thrombectomy (MT) alone with MT + alteplase which showed that MT alone was more cost-effective based on the Direct-MT trial [14, 37]. However, the indirect costs were not considered in the existing research [14]. Our study of AIS considered the evaluation of indirect costs for the first time among the AIS patients. Indirect costs were extracted from the disability-adjusted life years (DALYs) from published IS literature and discounted them to 2023 in the life expectancy scenario [22]. We found the indirect costs on ICER was not as sensitive as that of direct treatment costs, and did not affect the economic evaluation results of EVT alone.

As is well-known, the treatment time window was one of the important factors affecting the choice of treatment for patients with AIS [38, 39]. A Chinese study evaluated clinical outcomes and healthcare costs of different time windows of EVT among AIS patients, which was most cost-effective in 1–2 h after the stroke onset compared with other time windows (5–6 h) among patients choosing EVT [40]. From the pooled results of the meta-analysis which we extracted clinical outcomes, we found that the median time from stroke onset to randomized allocation of EVT alone was 134 min, and the median time for IVT plus EVT was 144 min [19]. In the future, the economic evaluation of AIS treatment could further explore the cost-effectiveness of EVT and IVT under the influence of the time windows, and we would like to further optimize the methodology.

Our study obtained model cost data from real-world Chinese databases, which provided the most reliable regional economic evaluation evidence of AIS treatment. Based on the HIS, we found that operative materials cost was a large part of the direct treatment costs, which could be considered future cost-effectiveness studies on surgical materials. Our study had several limitations. First, even though the clinical results from the meta-analysis of six RCTs were more valuable than those extracted from a single RCT, there were still trial bias. In the meta-analysis, the median age difference between the two groups was only one year. But patients who chose complex treatment plans were younger or stronger than who chose single treatment plans [41]. If we could follow up patients with AIS, we would be able to achieve a prospective cohort study in the further. Second, we assumed that patients with recurrent stroke who received the same treatment as those with initial stroke would have the same outcome. In general, recurrent stroke patients get worse with a higher mortality than those with first-time stroke. Finally, our conclusions reflected region-specific data, which maybe modified for other regions in China.

## Conclusions

This study indicated that EVT alone was more cost-effective compared with EVT + IVT based on the data from the real-world evidence and six RCTs’ meta-analysis for AIS in Shandong province, China. Although the conclusions were similar to most existing research recommendations, expansion in other regions still should be re-evaluated.

### Electronic supplementary material

Below is the link to the electronic supplementary material.

Supplementary Material 1

Supplementary Material 2

## Data Availability

Parameter data is provided in the manuscript. Data of direct medical costs in HIS system is confidential. The decision model can be obtained from the corresponding author with reasonable request and provided for editorial and peer review.
