# Supplementary material for: Cost-effectiveness of endovascular treatment for acute ischemic stroke in China: evidence from Shandong Peninsula
Source: Health Econ Rev. 2024 Jun 5;14:37. doi: 10.1186/s13561-024-00513-7 (PMC11154974; doi:10.1186/s13561-024-00513-7)
Supplement: Supplementary file 2 — Supplementary Material 2 [file 13561_2024_513_MOESM2_ESM.docx]

Supplement File 2

Supplementary Table1. Statistical description of the direct treatment costs in the affiliated hospital of Qingdao University.

|  | EVT alone  (n=3419) | EVT+IVT  (n=30) |
| --- | --- | --- |
| Demographic characteristics |  |  |
| Age | 66(60-71) | 66.5(53-71) |
| Gender |  |  |
| Male | 2704(79.09%) | 17(56.67%) |
| Female | 715(20.91%) | 13(43.33%) |
| Costs |  |  |
| Direct treatment costs | 71329.86(69890.76-72768.96) | 108463.6(86765.9-130161.3) |
| Operative treatment | 6543.83(6447.18-6640.49) | 10041.25(8220.18-11862.32) |
| Operative materials | 43751.21(43107.73-44394.69) | 52879.26(43079.18-62679.34) |
| Medicine | 6984.55(6660.73-7308.37) | 13285.07(10193.78-16376.36) |
| Nursing | 844.83(737.18-952.48) | 3035.45(1346.24-4724.66) |

Age data is median (IQR); Gender data is n (%); Costs data is mean(95%).
